# The Effect of Different Growth Stages of Black Chokeberry Fruits on Phytonutrients, Anti-Lipase Activity, and Antioxidant Capacity

**DOI:** 10.3390/molecules27228031

**Published:** 2022-11-19

**Authors:** Dorota Sosnowska, Dominika Kajszczak, Anna Podsędek

**Affiliations:** Institute of Molecular and Industrial Biotechnology, Faculty of Biotechnology and Food Sciences, Lodz University of Technology, Stefanowskiego 2/22, 90-537 Łódź, Poland

**Keywords:** black chokeberry, ripening stages, anti-lipase activity, antioxidant capacity, phenolic profile, macronutrients

## Abstract

The present study investigated the nutrients, biologically-active compounds, as well as antioxidant and anti-lipase activities of chokeberry fruits across four different stages of development, from the unripe green to mature black forms. The highest content of total phenolics (12.30% dry weight (DW)), including proanthocyanidins (6.83% DW), phenolic acids (6.57% DW), flavanols (0.56% DW), flavonols (0.62% DW), and flavanones (0.10% DW), was observed in unripe fruits. The unripe green fruits were also characterized by the highest content of protein (2.02% DW), ash (4.05% DW), total fiber (39.43% DW), and chlorophylls (75.48 mg/100 g DW). Ripe black fruits were the richest source of total carotenoids (8.53 mg/100 g DW), total anthocyanins (2.64 g/100 g DW), and total sugars (33.84% DW). The phenolic compounds of green fruits were dominated by phenolic acids (above 83% of the total content), the semi-mature fruits by both phenolic acids and anthocyanins (90%), while the mature berries were dominated by anthocyanins (64%). Unripe fruits were the most effective inhibitor of pancreatic lipase in triolein emulsion, scavenger of 2,2’-azinobis-(3-ethylbenzothiazolin-6-sulfonic acid) radical cation, and reducer of ferric ion. Biological activities were mainly correlated with total proanthocyanidins and total phenolics. Considering their strong anti-lipase and antioxidant activities, unripe chokeberry fruits may have potential applications in nutraceuticals and functional foods.

## 1. Introduction

Fresh, unprocessed black chokeberry fruits (*Aronia melanocarpa*) and fruits processed into juices, syrups, jams, teas, dried fruits, or dietary supplements display potential health benefits, including antioxidant, hepatoprotective, antihyperlipidemic, anticancer, anti-inflammatory, antimicrobial, anti-diabetic, and anti-obesity properties [1,2,3,4,5]. The above pro-health properties are mainly related to the very high content of phenolic compounds, including proanthocyanidins, anthocyanins, flavonols, and phenolic acids. Polymeric procyanidins were identified as the major class of polyphenols and represent about 60–66% of the fruit’s phenolic compounds [2,6]. Additionally, procyanidins with a polymerization degree above ten accounted for 82–99.9% of the total procyanidins of black chokeberries [7,8]. Anthocyanins account for about 18–23% of total phenolics and are represented mainly by glycosides of cyanidin, such as 3-galactoside, 3-arabinoside, 3-xyloside, and 3-glucoside [6,7,9,10]. Quantitatively, the next groups of phenolic compounds present in ripe *A. melanocarpa* fruits are the phenolic acids, followed by flavonols, which constitute about 11% and 4% of total phenolics, respectively [6]. Chlorogenic and neochlorogenic acids are dominant compounds among the phenolic acids, while flavonols are dominated by quercetin derivatives [9,11,12]. The qualitative and quantitative composition of phenolic compounds in the mature chokeberry fruit is well recognized and depends, among other things, on the variety, environmental factors, and agricultural practice [7,10,13,14,15]. In addition, the antioxidant potential of ripe chokeberries has been the subject of many studies [8,9,10,13,15,16,17,18]. The cited studies demonstrated the ability of *A. melanocarpa* fruit components to scavenge stable synthetic radicals such as 1,1-diphenyl-2-picrylhydrazyl (DPPH) and 2,2’-azinobis-(3-ethylbenzothiazoline-6-sulfonic acid) (ABTS), and also their scavenging activity against hydroxyl and peroxyl radicals. Moreover, the activity of scavenging nitric oxide and superoxide anion was demonstrated, as well as inhibition of lipid peroxidation in the liver. On the other hand, recent research has pointed to unripe chokeberry fruits as a valuable source of biologically-active compounds with high antioxidant activity [19,20]. Gralec et al. [19] observed the highest content of total phenolics, procyanidins, and flavonoids, as well as a higher scavenging activity of peroxyl and DPPH radicals for green and pink tinted green fruits than for red and purple-black fruits of Nero cultivar. Similar changes during *A. melanocarpa* fruit development for three other varieties, Viking, McKenzie, and Kingstar K1, were shown by Yang et al. [20]. The red tip stage of fruits exhibited a higher scavenging activity against ABTS and DPPH radicals, as well as the content of total phenolics and flavonoids being higher than that in red or dark purple fruits. In both studies, only the concentration of anthocyanin pigments reached the highest level in mature fruits.

Despite significant changes in the total content of phenolic compounds, as well as their different subgroups, the phenolic profile during the development of chokeberries has not been described so far. To the best of our knowledge, there are also no data on changes in lipophilic pigments during the ripening of chokeberry fruits. Therefore, the aim of this study was to perform a comprehensive spectrophotometric and chromatographic analysis of the composition of various bioactive phytocompounds, such as chlorophylls, carotenoids, and especially phenolic compounds, during the development of *A. melanocarpa* fruits, ranging from unripe green to ripe black fruits. Moreover, there have been no reports on the influence of the unripe and semi-mature developmental stages of chokeberry fruits on biological properties other than antioxidant activity, especially in minimizing the risk of metabolic diseases. Our previous studies showed the high inhibitory activity of mature chokeberry fruits against pancreatic lipase in different in vitro models [11,21,22]. In view of this, the effect of chokeberries at different development stages on pancreatic lipase activity in emulsified triolein has been studied. Since the biological activity of these fruits is related to their antioxidant properties, the scavenging effect toward ABTS radicals and the ferric reducing activity of berries at four stages of development were also studied. Comprehensive analysis of chokeberry fruits at various stages of maturity also included determination of the main macronutrients (protein, fat, ash, sugars, and dietary fiber).

## 2. Results and Discussion

### 2.1. Macronutrients of Chokeberry Fruits at Different Stages of Development

Despite the various chemical and biological evaluations performed for black chokeberry fruits, there have been very limited studies on the nutrient composition at various stages of fruit development [19,20]. This is an important issue, because during growth and ripening of fruits, a series of biochemical reactions are switched on that lead to the production or degradation of both primary and secondary plant metabolites. To date, some studies have been conducted to determine a number of biochemical features of ripe chokeberry fruits and after the ripening period, in order to select the optimal date for harvest [23,24,25]. In the present work, the subject of the research was unripe green, semi-mature purple, and ripe black *A. melanocarpa* fruits, picked every month from May to August, 2018. Their appearance, weight, and dry matter are shown in Table S1. The weight of 100 fruits increased by 2.1 times in June (S2), 10.3 times in July (S3), and 10.7 times in August (S4) compared to the weight of the fruits picked on 20 May (S1). The weight of 100 ripe chokeberry fruits was 94.4 g and was comparable to the weight of Nero and Viking cultivar fruits (92–102 g/100 fruits), which was determined by other authors [14,24]. Jeppson and Johansson [23] reported that the weight of 100 berries of chokeberry Aron, Nero, and Viking cultivars increased significantly from 75 g to 99 g between 14 and 22 August. During ripening, the dry matter increased considerably (by 38.6%) in the initial stages of development (from May to June), then decreased by 32.5% in July, and remained fairly constant in August (Table S1). Mazilu et al. [26] observed an increase by about 33% in the dry matter content of chokeberry fruits from 27 July to 22 August in Romania. On the other hand, the changes in the dry matter content of fruits from South Korea harvested in June and August varied, depending on the variety, and ranged from 14.60% to 48.60% [20]. Many studies on the qualitative analysis of mature chokeberries revealed that the dry matter content was in the range of 15.50 to 54.08 g/100 g [14,24,27,28].

The contents of ash, protein, fat, total fiber, and titratable acidity of chokeberry fruits at the various stages of development are shown in Table 1. The obtained values are given in grams per 100 g of fruit dry weight (DW), due to the large variation in the dry matter content, both in the present and published studies.

Statistically significant differences (*p* < 0.05) in the content of protein and sugars between chokeberry fruits at different developmental stages were observed. During the development of chokeberry fruits from May to August, the ash, protein, and total fiber content decreased, while the total sugars content increased. In the available literature, there are no data on changes in ash, protein, fat, and fiber content during the growth and maturation of chokeberry fruits. On the other hand, an increase in sugar content and slight changes in total acidity during fruit ripening of three chokeberry cultivars (Viking, McKenzie, and Kingstar K1) were confirmed by Yang et al. [20]. The sugar content (as the sum of fructose, glucose, and sorbitol) determined by Yang et al. [20] increased from 4.6 to 6.5 times in the period from June to August; while in our research, the fruits harvested in August (S4) contained nearly twice as much sugar as the fruits picked in May (S1).

The unripe green fruits (S1) were characterized by the highest content of ash (4.05 g/100 g DW), protein (2.02 g/100 g DW), total fiber (39.43 g/100 g DW), as well as soluble and insoluble dietary fiber (0.84 and 38.59 g/100 g DW, respectively). Semi-mature purple fruits (S3) contained the most fat (5.10 g/100 g DW), while ripe black fruits (S4) were the most abundant in sugars (33.84 g/100 g DW). Generally, fiber was the quantitatively dominant component in fruits at the three stages of development (S1, S2 and S3), followed by sugars. For comparison, fully ripe fruits (S4) were characterized by a higher sugar content than fiber content. Regardless of the fruit maturity stage, the insoluble fiber fraction (IDF) was the dominant fraction of fiber and constituted 97.0–97.9% of the total fiber content. During fruit development from May (S1) and June (S2) to August (S4), the content of IDF significantly (*p* < 0.05) decreased, from 38.59 to 26.18 g/100 g DW. The main component of the IDF fraction and, simultaneously, of the total fiber was Klason lignin, with the content ranging from 17.45 to 22.62 g/100 g DW, and constituting from 50.22 to 68.65% of the total IDF content (Figure S1). In the case of the SDF fraction, a decrease in its content was recorded in the period from May (S1) to July (S3) by 8.33%, followed by an increase in its concentration by 5.19%. Despite this, the green fruits harvested in May had the highest SDF content. In the SDF fraction, neutral sugars dominated (53.01–65.43%). According to Tanaka and Tanaka [29], mature black chokeberries contained about 36 g dietary fiber per 100 g fruit DW. This value is higher than that determined in the present study (26.99 g/100 g DW). IDF is the fraction of DF which affects consistency and stool weight and consequently reduces the intestinal transit time [30]. SDF, after ingestion, is fermented by gut microbiota, leading to the production of short chain fatty acids, mainly acetate, propionate, and butyrate, with various beneficial health effects [31]. The recommended daily dietary fiber intake for human adults is 25 g, and 70–80% of this amount should be insoluble [32].

### 2.2. Antioxidants of Chokeberry Fruits at Different Stages of Development

The content of bioactive phytocompounds possessing antioxidant properties at various stages of chokeberry fruit development is presented in Table 2.

The total content of chlorophylls systematically decreased during ripening and was about 35 and 25 times lower in ripe black fruits (S4) compared to unripe green fruits S1 and S2, respectively. Moreover, at all stages of fruit development, chlorophyll *a* dominated, with the ratio to chlorophyll *b* ranging from 1.65 at the S2 stage to 2.64 at the S4 stage. On the contrary, during fruit growth, an almost five-times increase in the content of carotenoids was observed (from 1.70 to 8.35 mg/100 g DW). The carotenoid content of the fresh chokeberries was found to be 4.86 mg/100 g, including 1.67 mg β-carotene, 1.22 mg β-cryptoxanthin, and 1.30 mg violaxanthin [33]. To the best of our knowledge, there are no data on changes of both chlorophyll and carotenoid pigments during the ripening of *A. melanocarpa* fruits. Chung et al. [34] showed a 4.6 fold reduction in the content of total chlorophylls during the ripening of highbush blueberries from pale green to dark purple color. Thus, the degradation of chlorophylls is also a major factor that contributes to the change in color of the fruit as it ripens. Loss of chlorophylls is likely due to enzymatic degradation and oxidation processes and a significant increase in non-photosynthetic pigments such as anthocyanins and carotenoids [35]. Unlike the chlorophyll and carotenoid pigments, anthocyanins were determined only in semi-mature purple (S3) and mature black (S4) fruits, and their content increased from 1.29 to 2.64 g/100 g DW. The changes found are consistent with those reported by Gralec et al. [19], because the content of anthocyanins increases from about 1 g/100 g DW in the fruits harvested in July to 2–3 g/100 g DW in the August fruits. Yang et al. [20] also reported that the total anthocyanins increased about 6 times when the chokeberry color changed from red to dark purple. The analysis of anthocyanins content confirmed that the analyzed ripe black *A. melanocarpa* fruit was a rich source of these pigments. Mature chokeberry fruits contained total anthocyanins in the range of 0.64–3.92 g/100 g DW [3,26,33].

The analysis of the obtained data confirmed the decrease in the content of phenolic compounds, such as phenolic acids, flavanols, flavonols, and flavanones, during the fruit development, but with an increase in anthocyanins. Changes in the content of all groups of phenolic compounds were statistically significant (*p* < 0.05). Overall, in the period from May to August, the concentration of the sum of phenolic compounds decreased almost twice, flavonols and flavanones more than three times, and the content of flavanols decreased by as much as 11 times. Mature chokeberry fruits contained total polyphenols in the range of 0.1–19.7 g per 100 g DW, while the total proanthocyanidins varied from 0.2 to 10.7 g/100 g DW [3,5,7]. The contents obtained in the presented study for ripe fruit are within the scope of the literature data (Table 2). The content of total phenolics and total proanthocyanidins decreased as the chokeberry fruit ripened. From May to August, the total phenolics decreased near three times (from 12.30 to 4.23 g/100 g DW) and the total proanthocyanidins more than seven times (from 6.83 to 0.94 g/100 g DW). Similarly to our results, Yang et al. [20] reported that the content of phenolic compounds at the red tip stage of *A. melanocarpa* fruit was significantly higher than that at the red and dark purple stage. Total phenolics decreased about two times from June to July, and about 2.7 times from June to August. The present results are also in agreement with the findings of Gralec et al. [19], with the highest total phenolics in green fruits (about 20 g/100 g DW) and the lowest in ripe purple-black fruits (about 10 g/100 g DW). Moreover, the highest content of total proanthocyanidins was observed for unripe fruits (10–15 g/100 g DW), but this declined to 1–8 g/100 g DW during fruit development from May to August. It is also worth emphasizing the reduction in the contribution of total proanthocyanidins to the total phenolics, namely from 56–58% in green fruits (S1–S2) to 27 and 22% in purple and dark fruits, respectively. A higher contribution of proanthocyanidins in the pool of phenolic compounds in mature fruit was reported by Teleszko and Wojdyło [6] and Oszmiański and Wojdyło [16]. Proanthocyanidins accounted for 60–66% of the total content of phenolic compounds. These discrepancies may have resulted from the use of different methods for the determination of proanthocyanidins. In the works cited, the content was determined using the HPLC method, after the thiolysis or phloroglucinolysis process. In the present study, these compounds were analyzed using the spectrophotometric method, after depolymerization in acidified butanol.

The phenolic composition of ripe chokeberry fruits has been determined in many studies, while the phenolic profile of unripe fruits was determined for the first time in this work [2]. Determination of the phenolic compounds profile of chokeberry fruits at various stages of development was also carried using the UPLC-MS method, and the results are summarized in Table 3.

Statistically significant differences (*p* < 0.05) in the total content of phenolic compounds and their individual subgroups, such as phenolic acids, flavanols, flavonols, flavanones, and anthocyanins were observed among chokeberry fruits at different developmental stages. Thirty seven phenolic compounds, including twelve phenolic acids, eleven flavonols, seven flavanols, four anthocyanins, and three flavanones were determined at the four ripening stages of chokeberry fruit. The phenolic acids in chokeberry fruits, regardless of their degree of ripeness, were dominated by 3-caffeoylquinic acid and 5-caffeoylquinic acid. The concentration of these acids during the growth of fruits from S1 to S4 decreased four and five times, respectively. The sum of their contents accounted for 90.7–96.9% of all determined phenolic acids. It should also be emphasized that semi-mature (S3) and mature (S4) fruits did not contain all of the seven compounds found in unripe green fruits (S1 and S2). These included the three isomers of caffeoylquinic acid, feruoylquinic acid, *p*-coumaroylquinic acid, 3,5-dicaffeoylquinic acid, and vanillate hexoside. Flavonol compounds were represented by quercetin, kaempferol, and isorhamnetin derivatives, and their total content decreased from 623.55 to 199.51 mg/100 g DW of fruit harvested in May (S1) and August (S4), respectively. The results of the composition of flavonols demonstrated the predominance of quercetin 3-galactoside and 3-rutinoside in fruits at all developmental stages. Their total content accounted for 44.7–57.1% of the total flavonols. Unripe green fruits (S1) did not contain quercetin 3-*O*-dihexoside and quercetin 3-*O*-robinoside, while unripe fruits harvested one month later contained (S2) quercetin 3-*O*-dihexoside and quercetin 3-*O*-rutinoside. Kaempferol 3-*O*-rutinoside was found only in fruits harvested in May (S1), and kaempferol 3-*O*-sophoroside was not present in ripe black fruits. The results of the UPLC-MS analyses showed an approximately tenfold decrease in flavanols content in semi-mature (S3) and mature (S4) fruits compared to unripe fruits (S1 and S2). Procyanidin C1 and procyanidin tetramer were quantitatively the dominant flavanols in fruits harvested in May (S1), and procyanidn C1 and procyanidin B2 in chokeberries from June (S2). Only one flavanol, the procyanidin trimer, was identified in semi-mature purple fruits (S3), and with only two procyanidin trimers in ripe black fruits (S4). Moreover, with the increased time of fruit ripening, a decrease in the content of two polyphenols from the flavanones group, eriodictyol hexoside and eriodictyol 7-glucoside, was observed. The presence of naringinin hexoside was only confirmed in ripe black fruits (S4). Contrary to the aforementioned groups of phenolic compounds, the content of anthocyanins increased during maturation, causing the expected changes in their color. Four cyanidin derivatives were determined in chokeberry fruits, of which cyanidin 3-arabinoside and 3-galactoside dominated in both semi-mature (S3) and mature (S4) fruits.

### 2.3. In Vitro Inhibition of Pancreatic Lipase by Chokeberry Fruits at Different Stages of Development

Pancreatic lipase (EC 3.1.1.3; triacylglycerol acyl hydrolase) produced by the pancreatic acinar cells hydrolyzes triglycerides, mainly into 2-monoacylglycerols and free fatty acids, and it is responsible for the hydrolysis of 50–70% of total dietary fats in the intestinal lumen [36]. Inhibition of pancreatic lipase, which splits triacylglycerols into absorbable monoglycerol and fatty acids, is an important strategy in the treatment of obesity. *A. melanocarpa* fruit was found to strongly inhibit the activity of pancreatic lipase in different in vitro assay systems [11,21,22,37]. The effect of chokeberry fruits at different stages of development (S1–S4) on the porcine pancreatic lipase activity was determined in vitro using triolein emulsion as a lipid substrate. The analyzed chokeberry fruits were also assessed using kinetic studies, to determine the type of pancreatic lipase inhibition. The results of the calculated IC_50_ value, based on Figure S2, and the calculated Michaelis–Menten kinetic parameters, based on the determined Lineweaver–Burk plots (Figure S3), such as Michaelis constant (Km), maximum speed (Vmax), and inhibition constant (Ki) are summarized in Table 4. All analyzed chokeberry fruits showed the ability to inhibit the activity of porcine pancreatic lipase in a dose-dependent manner (Figure S2). Based on the IC_50_ and K_i_ values, it was found that unripe fruits (S1 and S2) were the most effective lipase inhibitors. Their inhibitory activity was significantly higher (*p* < 0.05) than that of semi-mature purple fruits (S3) and ripe black fruits (S4). For comparison, ripe fruits (S4) showed about a 7.5 times higher IC_50_ value and about a 15 times higher K_i_ value than the most active fruits picked in June (S2) and May (S1), respectively. For comparison, the IC_50_ for Orlistat (a drug used to treat obesity) was only 0.34 ± 0.01 µg/mL (data not shown). Thus, the analyzed fruits showed much lower activity than orlistat. The weaker inhibitory effect of fruit extracts, including chokeberry components, on pancreatic lipase, as compared to that for orlistat, has been observed by others [11,21,38,39]. Similarly, unripe fruits of *Cudrania tricuspidata* [40] and unripe strawberries [41] showed a higher ability to inhibit pancreatic lipase activity than ripe fruits, which were characterized by a 2–3 times higher content of phenolic compounds. In the above studies, *p*-nitrophenyl butyrate and 4-methylumbelliferyl oleate were used as substrates, respectively. In the present study, the anti-lipase activity of chokeberry fruits at different stages of development was mainly correlated with total proanthocyanidins and phenolics (R = 1.00), and to a lesser extent with total flavanols (R = 0.97), total phenolic acids (R = 0.91), flavonols (R = 0.81), and flavanones (R = 0.71). The correlation between phenolic compounds content, especially tannin-like components, and inhibitory activity against lipase was demonstrated by others [11,42,43,44,45].

Regardless of the degree of ripeness, all tested chokeberry fruits showed a mixed type of inhibition against pancreatic lipase, because the lines of lipase inhibition did not intersect the X-axis or the Y-axis. Moreover, the values of the maximum velocity (*V*max) and the Michaelis–Menten constant (*Km*) increased with the concentration of inhibitors (expressed in fruit equivalents) in the reaction mixture.

**Figure 1:**
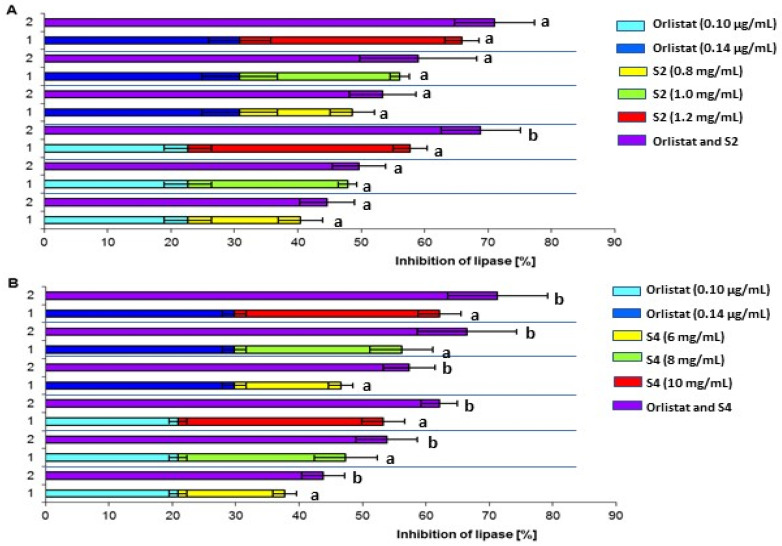
Percentage lipase inhibition by unripe fruits (S2) and orlistat (O)—(**A**), and ripe fruits (S4) and orlistat—(**B**) at various concentrations; 1—inhibition of lipase by single inhibitors; 2—inhibition of lipase by mixture of inhibitors; values are means ± SD (*n* = 3). Different letters denote significant differences (*p* < 0.05) between the sum of the percentage inhibition caused by individual inhibitors (1) and the inhibition caused by a mixture of these inhibitors (2).

In the next part of the present work, the possibility of combined use of orlistat and the most active chokeberry fruit sample (S2), with IC_50_ = 1.97 mg/mL, was investigated. A ripe chokeberry sample (S4) was used for comparison, because when ripe, fruit can be part of our diet. Orlistat can cause serious side effects, so dietary constituents administered in combination with orlistat could be a more safe agent to prevent and treat obesity [46]. The research was carried out for three concentrations of chokeberry and two orlistat samples, which were selected on the basis of experimental results conditioning the inhibition of lipase inhibition up to 30%. The obtained results are presented in Figure 1. A comparison of the total activity of the unripe fruit sample (S2) and orlistat with the anti-lipase activity of their mixture is shown in Figure 1A. Statistically significant differences (*p* < 0.05) were only observed for one combination out of the six variants, in which the ratio of the fruit dose to orlistat was 12,000:1. With a lower excess of fruit sample compared to orlistat (from 5700:1 to 10,000:1), the differences were not statistically significant, despite the higher degree of lipase inhibition by the mixture. In the case of the mixture of ripe chokeberry fruits (S4) with orlistat, statistically significant differences were found in all concentration variants (Figure 1B). It should be emphasized that the ratio of the concentration of ripe chokeberry fruit sample to orlistat ranged from 43,000:1 to 100,000:1.

Previous research showed a lack of statistically significant differences between the anti-lipase activity of orlistat and chokeberry fruit crude, phenolic-rich, and proanthocyanidins-rich extract mixtures compared to the activity of the sum of individual extracts and orlistat [11,47]. In the cited studies, the concentration ratio of the extracts used to orlistat was in the range 26.500:1 to 74,000:1 for crude extract, 6000:1 to 14,000:1 for phenolic-rich extract, and from 1200:1 to 2600 for proanthocyanidins-rich extract.

### 2.4. Antioxidant Capacity of Chokeberry Fruits at Different Stages of Development

Mature black chokeberry fruits are known as a natural source of numerous compounds with antioxidant properties, such as vitamin C, vitamin E, carotenoids, and phenolic compounds [3]. In terms of their antioxidant potential, they are superior to other popular fruits [18,22,28,48]. The antioxidative activity of mature chokeberries was confirmed in various radical scavenging assays, together with the effects of transition metals on changes in the state of oxidation and the ability to inhibit lipid peroxidation in a variety of model systems [3]. Antioxidants may protect human cells from reactive oxygen species and reactive nitrogen species, whose generation is exacerbated under pathological conditions in different ways. Antioxidants can convert free radicals into non-radical compounds, break the chain reaction of lipid oxidation, inhibit pro-oxidative enzymes, and chelate metal ions [20].

**Figure 2:**
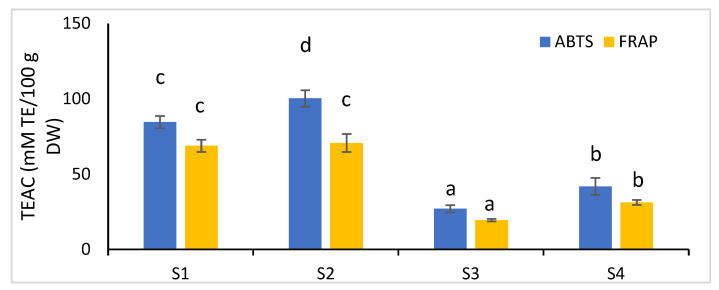
Antioxidant capacity of chokeberry fruit during ripening. S1—unripe green fruits, harvested 20 May; S2—unripe green fruits, harvested 20 June; S3—semi-mature purple fruits, harvested 20 July; S4—ripe black fruits, harvested 20 August; Different letters denote significant differences (*p* < 0.05) between dates of fruit harvest, *n* = 5.

The antioxidant capacity of chokeberry fruits at different stages of development was estimated as the scavenging potential toward ABTS^•+^ radical cation and the potential to reduce ferric to ferrous ion (FRAP method). The results, expressed as Trolox equivalents antioxidant capacity (TEAC), are shown in Figure 2. Among the analyzed fruits, significant differences (*p* < 0.05) were found in the antioxidant capacity, with the exception of the iron (III) ion reduction efficiency of unripe green fruits (S1 and S2). The TEAC value decreased during the ripening of chokeberry fruits from 84.49 to 41.77 mM TE/100 g DW and from 68.79 to 31.17 mM TE/100 g DW, using the ABTS and FRAP methods, respectively. The changes in the antioxidant activity during the fruit ripening process are consistent with other studies. Gralec et al. [19] observed the highest scavenging activity of peroxyl (ROO^•^) and DPPH^•^ radicals for green and pink tinted green fruits, as compared to that for red and purple-black chokeberries of the Nero cultivar. Similar changes during *A. melanocarpa* fruit development for three other varieties, Viking, McKenzie, and Kingstar K1, were shown by Yang et al. [20]. The red tip stage of fruits exhibited a higher scavenging activity against ABTS and DPPH radicals, as well as content of total phenolics and flavonoids, than that exhibited by red or dark purple fruits. In the present study, the scavenging effect toward the ABTS^•+^ cation radical of mature chokeberry fruits (S4) was five times lower than that shown by Samoticha et al. [49] but comparable to the TEAC value reported by Teleszko and Wojdyło [6]. Whereas, the TEAC values determined using the FRAP method were confirmed by these authors.

Similarly, Yang et al. [20] showed a positive high correlation (R > 0.97) between the content of phenolic compounds and flavonoids and the scavenging efficiency of ABTS and DPPH radicals for three cultivars of *A. melanocarpa* fruits at various stages of development (from red tip to dark purple stages). Moreover, the chlorogenic acid content and antioxidant activities were highly correlated (R > 0.9). On the other hand, the correlation between anthocyanin content and the antioxidant potential was negative (R = −0.780 and −0.877 for the DPPH and ABTS methods, respectively). Lower values of correlation coefficient (0.70–0.77) were found for the relationship between the content of total phenolics and the effectiveness of scavenging DPPH^•^ (DPPH-EPR method) and ROO^•^ radicals (ORAC method) for various stages of development of chokeberry fruits collected in the period from May to August [19]. The correlation between the different groups of phenolic compounds and antioxidant potential determined in the present study is shown in Table 5. The antioxidant properties of chokeberry fruits at various stages of development, determined using the ABTS and FRAP methods, were positively correlated to the highest degree with the content of total phenolics, proanthocyanidins, and flavanols (R > 0.90). A slightly lower correlation (R > 0.80) was found between the antioxidant activity and the content of phenolic acids (mainly 3- and 5-caffeoylquinic acids—Table 2). The antioxidant potential of the fruits tested was less correlated (R < 0.76) with the flavonols and flavanones content. In the present study, no correlation coefficient was determined for anthocyanins, due to them not being present in unripe fruits. 

## 3. Conclusions

Mature chokeberry fruits, although not consumed directly, are used in the production of many fruit preserves, food dyes, and fiber preparations. Despite the various chemical and biological evaluations performed for black chokeberry fruits, there is insufficient information about the chemical composition and activity of unripe and semi-mature fruits. The results obtained in the present study indicate that unripe green chokeberry fruits are also a good source of nutrients and antioxidants, and that they exhibit pancreatic lipase inhibition and antioxidant activity. In contrast, ripe fruits were only characterized by a higher content of carotenoids and sugars, as well as the presence of anthocyanins. We showed, not only quantitative but also qualitative, differences in the phenolic compounds at the four developmental stages of the fruit. To the best of our knowledge, there has been no previous study which evaluated the anti-lipase potential, the ability to reduce iron (III) ions, the content of lipophilic pigments (carotenoids and chlorophylls), and the composition of phenolic compounds in the process of growth and ripening of *A. melanocarpa* fruits. On the basis of these promising results, especially the strong anti-lipase and antioxidant activities, further research on the health-promoting properties of unripe fruits and their possible use in the production of functional food components and dietary supplements should be carried out in the future.

## 4. Materials and Methods

### 4.1. Standards and Reagents

Anthrone, 2,2′-azinobis(3-ethyl-benzthiazoline-6-sulphonic acid) (ABTS), bile from bovine and ovine, 3,5-dimethylphenol, 6-hydroxy-2,5,7,8-tetramethychroman-2-carboxylic acid (Trolox), galacturonic acid, glucose, iron(III) chloride, lipase (EC 3.1.1.3) from porcine type II, potassium persulfate, TRIS-base, 2,4,6-tris-2-pyridyl-*s*-triazine (TPTZ), orlistat, protease from *Bacillus licheniformis* (≥2.4 U/g), and triolein were obtained from Sigma-Aldrich (Steinheim, Germany). Acetonitrile (Merck, Germany) and formic acid (Sigma-Aldrich, Steinheim, Germany) were hyper grade for LC-MS. Boric acid, butanol, cooper acetate, dichloromethane, glacial acetic acid, hydrogen chloride, isooctane, petroleum ether, pyridine, sodium carbonate, sodium chloride, and sodium hydroxide (NaOH) were purchased from Chempur (Piekary Śląskie, Poland). Calcium chloride, Folin–,Ciocalteu reagent and ethanol were obtained from POCH (Gliwice, Poland); and acetone, n-hexane, methanol, and sulphuric acid from POCH BASIC (Gliwice, Poland). Reference compounds were obtained from Sigma-Aldrich (Steinheim, Germany) (β-carotene, (+)-catechin, gallic acid, kaempferol 3-glucoside, naringin, quercetin 3-rutinoside), and from Extrasynthese (Lyon, France) (5-caffeoylquinic acid, cyanidin 3-arabinoside, cyanidin 3-galactoside, cyanidin 3-glucoside, isorhamnetin 3-glucoside, isorhamnetin 3-rutinoside), as well as from PhytoLab (Vestenbergsgreuth, Germany) (3-caffeoylquinic acid, 4-caffeoylquinic acid, 3,5-dicaffeoylquinic acid, procyanidin B1, procyanidin B2, procyanidin C1, quercetin 3-glucoside). Ultrapure water (Simplicity^TM^ Water Purification System, Millipore, Marlborough, MA, USA) was used to prepare all solutions.

### 4.2. Plant Material

Black chokeberry fruits were harvested at four stages of development on a farm near Łódź, in the central region of Poland, in 2018. The unripe fruits were picked from the bush in May (S1) and June (S2), while semi-mature fruits were picked in July (S3). The last harvest was in August (S4), when the fruits were fully black colored. The first fruit harvest took place two weeks after the end of flowering of the chokeberry bush. The collected fruits were frozen overnight and freeze-dried for 2 days. The dry fruits were ground in a coffee grinder, and the powders were stored in tightly closed containers, without light. The harvest date, the appearance of fruits on the bush, and the freeze-dried fruits after crushing, as well as the weight of 100 fruits, are presented in Table S1.

### 4.3. Approximate Analysis

The elementary chemical composition of samples (dry matter, ash, protein, and fat contents) was determined using standard procedures [50]. Dry matter was estimated by drying at 105 °C to constant weight, the unit for the results was expressed in g/100 g of fruit fresh weight (FW) or g/100 g of freeze-dried fruit. Ash was determined through incineration of fruit samples in a muffle furnace at 600 °C for 6 h, crude protein by the Kjeldahl method (N × 6.25), and crude fat by Soxhlet extraction with hexane, and the results are shown as g/100 g of fruit dry weight (DW). Total acidity was determined by titration with NaOH (0.1 M) to an end point of pH 8.1 [51] and expressed as malic acid equivalents in g/100 g of DW. The total content of soluble sugars was determined in fruits deprived of fat with petroleum ether, according to Deng et al. [52]. Sugars were extracted from dry fruits four times with 80% ethanol for 10 min at room temperature in an ultrasonic bath, and the sugar content was determined using the method with an anthrone reagent in a sulfuric acid environment [53]. The sugar content is expressed as glucose equivalents in g/100 g of DW.

### 4.4. Determination of Dietary Fiber Components

The total dietary fiber (DF) content of dry chokeberry fruit was analyzed according to the procedure described by Gouw et al. [53]. Total fiber concentration was determined as the sum of soluble fiber (SDF) and insoluble fiber (IDF). SDF concentration was the sum of uronic acids (UA) and neutral sugars (NS), determined by spectrophotometric methods. On the other hand, the concentration of IDF was the sum of UA, NS, and the mass of Klason lignins (KL), which was quantified gravimetrically after subtracting the ash content. UA was determined by the 3,5-dimethylphenol reagent method, NS was determined with anthrone reagent, and their contents were expressed as galacturonic acid and glucose equivalents, respectively. The content of total fiber and all components of the SDF and IDF fractions was expressed as g/100 g of DW.

### 4.5. Extraction and Analysis of Total Carotenoids

Dry black chokeberry fruits (0.3 g) were extracted three times with 20 mL of hexane on a magnetic stirrer for 15 min. Absorbance of pooled organic layers was measured at 450 nm against the hexane, and the total carotenoids content was expressed as milligrams of β-carotene equivalents per 100 g of DW.

### 4.6. Extraction and Analysis of Total Chlorophylls

The unripe black chokeberry fruits (0.3 g) were extracted three times with 20 mL of 80% acetone on a magnetic stirrer for 15 min. On the other hand, semi-ripe and ripe chokeberry fruits (0.3 g), due to the presence of anthocyanin pigments, were extracted with dichloromethane under the same conditions as the unripe fruits. The pooled dichloromethane fractions were then evaporated to dryness in a vacuum evaporator, and the dry residue was dissolved in 80% acetone (3 mL). The absorbance of the acetone solutions was measured at 645 and 663 nm against 80% acetone. The concentrations of chlorophyll *a*, chlorophyll *b,* and total chlorophyll were calculated using the equation described by Rajalakshmi and Banu [54] and expressed as mg/100 g of DW.

### 4.7. Extraction for Measurement of Phenolic Compounds, Antioxidant Potential, and Anti-Lipase Activity

Dry black chokeberry fruits (5.0 g) were extracted twice with 200 mL of 70% ethanol on a magnetic stirrer at room temperature for 30 min. After centrifugation at 5000 rpm for 10 min, the combined supernatants were evaporated at 40 °C under reduced pressure, in order to remove ethanol to a final volume of 50 mL (equivalent to 0.1 g of freeze-dried fruit/mL). The extracts were stored at −24 °C for further studies.

### 4.8. Quantification of Phenolic Compounds Using Spectrophotometric Methods

Total phenolics were measured using Folin–Ciocalteu reagent, as described in our previous work [22]. The absorbance of reaction mixtures was measured at 760 nm after 20 min incubation at ambient temperature, and the content of total phenolics was expressed as gallic acid equivalents (GAE). The total proanthocyanidins were determined after their acid depolymerization to the corresponding anthocyanidins, as described by Rösch et al. [55], and was expressed as cyanidin equivalents (CYE) using the molar absorbance of ε = 17,360 L/mol* cm and molecular weight of 287 g/mol. The total anthocyanin content was measured using the pH differential method, as described previously [56], and expressed as cyanidin 3-glucoside equivalents (CGE) using the molar absorbance of ε = 26,900 L/mol* cm and molecular weight of 449.2 g/mol. The content of all groups of phenolic compounds was expressed as g/100 g of DW.

### 4.9. Determination of Individual Phenolic Compounds Using UPLC/Q-TOF-MS Analysis

The qualitative and quantitative composition of phenolic compounds was estimated using an Acquity ultra-performance liquid chromatography (UPLC) system coupled with a quadruple-time of flight mass spectrometry (Q-TOF-MS) instrument (Waters Corp., Milford, MA, USA) equipped with an electrospray ionization (ESI) source. Chromatographic separation was performed using an Acquity HSS T3 C18 column (150 mm × 2.1 mm, 1.8 µm; Waters, Milford, MA, USA), separation column temperature 30 °C, flow rate 0.45 mL/min, and injection volume 5 µL. The mobile phase was (A) 0.1% formic acid (*v*/*v*) and (B) acetonitrile (B) with gradient elution of 99% A (0 min), 65% A (12 min), 0% A (12.5 min), and 99% A (13.5 min). The conditions of MS/MS were electrospray negative mode, desolvation temperature 250 °C, desolvation gas flow of 600 L/h, cone voltage of 45 V, capillary voltage of 2.0 kV, and collision energy 50 V [57]. Leucine enkephalin was used as a lock mass. The instrument was controlled by Mass-Lynx^TM^ V 4.1 software. Phenolic compounds were identified using their UV-Vis characteristics and MS and MS^2^ properties using data gathered in house and from the literature. Calibration curves were run for the external standards: (+)-catechin, procyanidin B1, B2, and C1, 3-caffeoylquinic acid, 4-caffeoylquinic acid, 5-caffeoylquinic acid, 3,5-dicaffeoylquinic acid, gallic acid, quercetin 3-rutinoside, quercetin 3-glucoside, kaempferol 3-glucoside, isorhamnetin 3-rutinoside, isorhamnetin 3-glucoside, cyanidin 3-galactoside, cyanidin 3-glucoside, cyanidin 3-arabinoside, and naringin.

### 4.10. In Vitro Antioxidant Activity Assays

The antioxidant capacity of ethanol extracts obtained from chokeberry fruits was determined using ABTS radical cation (ABTS) scavenging activity and as ferric reducing antioxidant power (FRAP), according to the methods described by Podsędek et al. [22]. The antioxidant capacity of fruits was expressed as mM Trolox equivalents (TE) per 100 g DW of fruit.

### 4.11. Pancreatic Lipase Activity Assay

Immediately before analysis, 1.8 mL of 20 mM Tris-base buffer (pH 7.4, containing 150 mM NaCl and 1.3 mM CaCl_2_) was added to 2 mL of the extract, adjusted to pH 7.4 with 2 M NaOH, and then the volume was made up to 4 mL with the same buffer. Finally, 1 mL of stock solution of fruit sample was equivalent to 50 mg of freeze-dried chokeberry fruits. The pancreatic lipase activity was tested by measuring the fatty acids (with copper reagent) released from emulsified triolein according to the method described previously [11]. Inhibitory activity of chokeberry fruit extracts against pancreatic lipase was expressed as the IC_50_ values (half-maximal inhibitory concentration). The IC_50_ value was concluded from the graph of lipase inhibition (%) vs. the concentration of analyzed fruits per 1 mL of reaction mixture under assay conditions.

### 4.12. Kinetics of Inhibition against Pancreatic Lipase

The inhibition mode of the extract at different concentrations was measured with increasing concentrations of triolein as a substrate against lipase activity [11]. The inhibition type was determined by Lineweaver-Burk plot analysis, calculated according to Michaelis–Menten kinetics.

### 4.13. Combined Effect of Orlistat and Chokeberry Fruit Extracts

The various concentrations of orlistat (0.1, 0.14, and 0.18 µg/mL of reaction mixture) were combined with S2 stages concentration (0.8, 1.0, and 1.2 mg/mL of reaction mixture) or S4 stages concentration (6.0, 8.0, and 10.0 mg/mL of reaction mixture). The results were expressed as the percentage inhibition compared to the inhibitor-free control. A stock solution of orlistat was prepared in methanol at 0.1 mg/mL.

### 4.14. Statistical Analysis

Data from the present study were presented as the mean ± standard deviations of three replicates for each sample. Significant differences (*p* ≤ 0.05) between the means were evaluated by one-way ANOVA and Tukey’s HSD test. For comparison of the results of the total phenolics, total flavanols, total proanthocyanidins, inhibitory activity against pancreatic lipase, and antioxidant capacity, the coefficients of correlation were determined using a Pearson correlation test.

## Figures and Tables

**Table 1:** The content (g/100 g DW) of the main nutrients of chokeberry fruits at various stages of development.

Chemical Compounds	Stage of Fruit Development			
	S1	S2	S3	S4
Ash	4.05 ± 0.05 c	3.17 ± 0.06 b	2.43 ± 0.21 a	2.45 ± 0.03 a
Protein	2.02 ± 0.00 d	1.43 ± 0.01 c	0.89 ± 0.01 b	0.74 ± 0.01 a
Fat	3.35 ± 0.04 a	2.65 ± 0.13 b	4.10 ± 0.14 c	3.34 ± 0.05 a
Sugars	17.28 ± 0.54 a	19.30 ± 0.41 b	32.32 ± 0.83 c	33.84 ± 0.78 d
Total acidity	5.41 ± 0.00 b	4.18 ± 0.16 c	5.26 ± 0.04 ab	5.15 ± 0.09 a
Fiber total	39.43 ± 0.65 a	38.90 ± 0.77 a	33.72 ± 0.21 c	26.99 ± 0.62 b
SDF total	0.84 ± 0.02 a	0.83 ± 0.03 a	0.77 ± 0.01 b	0.81 ± 0.01 ab
IDF total	38.59 ± 0.65 a	38.07 ± 0.78 a	32.95 ± 0.60 c	26.18 ± 0.62 b

SDF—soluble dietary fiber, IDF—insoluble dietary fiber; S1—unripe green fruits, harvest 20 May; S2—unripe green fruits, harvest 20 June; S3—semi-mature purple fruits, harvest 20 July; S4—ripe black fruits, harvest 20 August; values are expressed as mean ± SD (*n* = 3); mean values within a row with different letters are significantly different at *p* < 0.05.

**Table 2:** The total content of phytocompounds in black chokeberry fruits at various stages of development.

Phytocompounds	Stage of Fruit Development			
	S1	S2	S3	S4
Carotenoids (mg β-carotene/100 g DW)	1.70 ± 0.28 a	1.27 ± 0.03 a	6.33 ± 0.11 b	8.35 ± 0.23 c
Chlorophylls total (mg/100 g DW)	75.48 ± 0.87 d	52.85 ± 0.50 c	5.03 ± 0.08 b	2.15 ± 0.12 a
Chlorophyll *a* (mg/100 g DW)	48.39 ± 0.56 d	32.94 ± 0.54 c	4.13 ± 0.02 b	1.56 ± 0.12 a
Chlorophyll *b* (mg/100 g DW)	27.12 ± 0.39 c	19.92 ± 0.27 b	0.91 ±0.10 a	0.59 ± 0.01 a
Phenolics total (g GAE/100 g DW)	12.30 ± 0.17 d	11.69 ± 0.49 c	2.70 ± 0.11 a	4.23 ± 0.22 b
Proanthocyanidins total (g CYE/100 g DW)	6.83 ± 0.36 b	6.76 ± 0.39 b	0.74 ± 0.05 a	0.94 ± 0.02 a
Anthocyanins total (g CGE/100 g DW)	-	-	1.29 ± 0.03 a	2.64 ± 0.10 b

GAE—gallic acid equivalents, CE—(+)-catechin equivalents, CYE—cyanidin equivalents, CGE—cyanidin glucoside equivalents; S1—unripe green fruits, harvest 20 May; S2—unripe green fruits, harvest 20 June; S3—semi-mature purple fruits, harvest 20 July; S4—ripe black fruits, harvest 20 August; values are expressed as mean ± SD (*n* = 3); mean values within a row with different letters are significantly different at *p* < 0.05.

**Table 3:** Content (mg/100 g DW) of phenolic compounds in black chokeberry fruit during ripening.

Compound	S1	S2	S3	S4
caffeoylquinic acid ^A^	40.07 ± 0.07	34.38 ± 0.08	15.37 ± 0.57	3.82 ± 0.18
3-caffeoylquinic acid	2381.86 ± 22.00	1956.56 ± 6.99	771.62 ± 1.40	591.33 ± 1.11
*p*-coumaroylquinic acid ^A^	138.89 ± 0.52	85.47 ± 0.09	42.46 ± 0.04	26.47 ± 0.01
vanillate hexoside ^B^	4.12 ± 0.16	27.52 ± 0.17	-	-
5-caffeoylquinic acid	3583.17 ± 24.08	2015.30 ± 1.34	854.75 ± 0.56	717.80 ± 0.94
4-caffeoylquinic acid	14.53 ± 0.62	17.37 ± 0.05	12.15 ± 0.08	11.23 ± 0.05
caffeoylquinic acid ^A^	40.70 ± 0.56	37.18 ± 0.02	-	-
feruoylquinic acid ^A^	49.26 ± 0.65	-	-	-
caffeoylquinic acid ^A^	180.16 ± 1.72	-	-	-
caffeoylquinic acid ^A^	36.93 ± 0.29	-	-	-
*p*-coumaroylquinic acid ^A^	95.80 ± 0.19	-	-	-
3,5-dicaffeoylquinic acid	8.61 ± 0.83	7.54 ± 0.51	-	-
***Total phenolic acids***	***6574.10 ± 41.32 d***	***4192.45 ± 13.20 c***	***1687.36 ± 1.44 b***	***1350.65 ± 2.26 a***
procyanidin B1	11.16 ± 1.65	46.08 ± 1.32	-	-
procyanidin trimer ^c^	50.40 ± 2.21	-	-	27.03 ± 0.58
(+)-catechin	81.38 ± 1.67	-	-	-
procyanidin B2	33.60 ± 2.68	137.21 ± 0.35	-	-
procyanidin trimer ^c^	-	-	79.72 ± 0.53	23.93 ± 0.46
procyanidin C1	194.49 ± 9.35	135.87 ± 0.38	-	-
tetramer procyjanidyny ^c^	192.06 ± 5.20	-	-	-
***Total flavanols***	***563.09 ± 10.87 d***	***319,17 ± 1.86 c***	***79.72 ± 0.53 b***	***50.96 ± 0.46 a***
quercetin 3-*O*-dihexoside ^D^	24.59 ± 0.14	22.93 ± 0.04	23.03 ± 0.07	12.96 ± 0.03
quercetin 3-*O*-dihexoside ^D^	-	-	7.04 ± 0.01	4.08 ± 0.02
quercetin 3-*O*-vicianoside ^D^	72.92 ± 0.53	43.29 ± 0.01	33.80 ± 0.03	17.76 ± 0.02
quercetin 3-*O*-galactoside ^D^	177.36 ± 4.23	116.64 ± 0.14	59.88 ± 0.44	51.66 ± 0.07
quercetin 3-*O*-robinoside ^D^	-	17.95 ± 0.04	17.38 ± 0.12	21.93 ± 0.50
quercetin 3-*O*-rutinoside	168.15 ± 1.13	96.85 ± 1.99	66.94 ± 0.90	49.06 ± 0.92
quercetin 3-*O*-glucoside	69.38 ± 2.10	49.98 ± 0.32	46.55 ± 0.05	37.48 ± 0.47
kaempferol 3-*O*-sophoroside ^E^	32.03 ± 0.18	11.93 ± 0.01	6.99 ± 0.28	-
kaempferol 3-*O*-rutinoside ^E^	54.56 ± 0.58	-	-	-
isorhamnetin 3-*O*-rutinoside	9.18 ± 0.14	7.99 ± 0.03	3.94 ± 0.01	2.51 ± 0.04
isorhamnetin 3-*O*-rhamnosylhexoside ^F^	15.39 ± 0.15	6.11 ± 0.03	5.74 ± 0.05	2.07 ± 0.03
***Total flavonols***	***623.55 ± 5.31 d***	***373.67 ± 2.43 c***	***271.29 ± 1.58 b***	***199.51 ± 0.87 a***
cyanidin 3-*O*-galactoside	-	-	668.97 ± 1.19	1271.98 ± 1.91
cyanidin 3-*O*-glucoside	-	-	65.52 ± 0.06	121.51 ± 0.12
cyanidin 3-*O*-arabinoside	-	-	995.67 ± 0.88	1375.07 ± 1.99
cyanidin 3-*O*-xyloside ^G^	-	-	63.79 ± 0.58	177.13 ± 2.05
***Total anthocyanins***	***-***	***-***	***1793.96 ± 2.61 a***	***2945.69 ± 2.94 b***
eriodictyol hexoside ^H^	30.79 ± 0.86	20.60 ± 0.30	7.95 ± 0.05	-
eriodictyol 7-glucuronide ^H^	74.78 ± 2.43	24.64 ± 0.03	29.86 ± 0.08	26.13 ± 0.01
naringinin hexoside ^H^	-	-	-	7.04 ± 0.08
***Total flavanones***	***105.57 ± 1.94 d***	***45.24 ± 0.27 c***	***37.79 ± 0.09 b***	***33.17 ± 0.08 a***
***Sum of phenolic compounds***	***7866.30 ± 35.97 d***	***4930.53 ± 17.62 c***	***3870.11 ± 5.02 a***	***4579.98 ± 3.94 b***

Values are expressed as mean ± SD (*n* = 3); ^A^: quantified as 5-caffeoylquinic acid equivalents, ^B^: quantified as gallic acid equivalents; ^C^: quantified as procyanidin C1 equivalents, ^D^: quantified as quercetin 3-*O*-glucoside equivalents, ^E^: quantified as kaempferol 3-*O*-glucoside equivalents ^F^: quantified as isorhamnetin 3-*O*-glucoside equivalents ^G^: quantified as cyanidin 3-O-glucoside equivalents, ^H^: quantified as naringin equivalents; S1—unripe green fruits, harvest 20 May; S2—unripe green fruits, harvest 20 June; S3—semi-mature purple fruits, harvest 20 July; S4—ripe black fruits, harvest 20 August; mean values within a row with different letters are significantly different at *p* < 0.05.

**Table 4:** Enzyme kinetics and IC_50_ value of chokeberry fruit during ripening against pancreatic lipase.

Fruit	Fruit Concentration (mg/mL)	K_m_ (mg/mL)	V_max_ (OD/min)	K_i_ (mg/mL)	Mode of Inhibition ^1^	IC_50_ (mg/mL) ^2^
S1	0	32.69	0.06	0.74	mixed	1.99 ± 0.04 a
	1.0	55.78	0.07			
	1.4	185.15	0.18			
	1.8	382.13	0.24			
S2	0	32.69	0.06	1.07	mixed	1.97 ± 0.12 a
	1.0	60.98	0.08			
	1.4	81.00	0.08			
	1.8	137.18	0.10			
S3	0	32.69	0.06	11.22	mixed	23.47 ± 0.01 c
	14	74.10	0.08			
	18	274.55	0.22			
	22	478.73	0.32			
S4	0	32.69	0.06	10.78	mixed	14.80 ± 0.29 b
	6	41.47	0.07			
	10	64.22	0.08			
	14	174.52	0.15			

^1^—inhibition mode was performed from double reciprocal plot; ^2^—values are expressed as mean ± SD (*n* = 3); S1—unripe green fruits, harvested 20 May; S2—unripe green fruits, harvested 20 June; S3—semi-mature purple fruits, harvested 20 July; S4—ripe black fruits, harvested 20 August; different letters in the IC_50_ column denote a statistical difference at *p* < 0.05.

**Table 5:** Pearson’s correlation coefficients (R) between the phenolics contents and antioxidant capacity and anti-lipase activity of chokeberry fruits with different stages of ripeness.

	ABTS	FRAP
Total phenolics ^1^	0.971	0.995
Proanthocyanidins ^1^	0.969	0.986
Phenolic acids ^2^	0.802	0.877
Flavanols ^2^	0.909	0.960
Flavonols ^2^	0.660	0.758
Flavanones ^2^	0.553	0.670

^1^—spectrophotometric methods; ^2^—HPLC methods.

## Data Availability

All data involved in this paper are given in the text or “Supplementary Materials”.

## Supplementary Materials

The following supporting information can be downloaded at: https://www.mdpi.com/article/10.3390/molecules27228031/s1, Figure S1: The percentage of soluble and insoluble fiber fraction in chokeberry fruit at various stages of development; Figure S2: Effect of concentration of chokeberry fruits at various stages of development (S1–S4) on the inhibition of pancreatic lipase; Figure S3: Lineweaver–Burk plots showing the impact of the control (without inhibitor) and chokeberry fruit at various stages of development (S1–S4) on the pancreatic lipase activity; Table S1: Description of the selected maturity stages for collection of chokeberry fruit.
