# Supplementary material for: The Effect of Different Growth Stages of Black Chokeberry Fruits on Phytonutrients, Anti-Lipase Activity, and Antioxidant Capacity
Source: Molecules. 2022 Nov 19;27(22):8031. doi: 10.3390/molecules27228031 (PMC9695515; doi:10.3390/molecules27228031)
Supplement: Supplementary file 1 [file molecules-27-08031-s001.zip › molecules-2036211-supplementary.pdf]

SUPPLEMNETARY MATERIALS

The effect of different growth stages of black chokeberry fruits on phytonutrients, anti-lipase activity, and antioxidant capacity

Dorota Sosnowska[1], Dominika Kajszczak[1] and Anna Podsędek[1]*

1 Institute of Molecular and Industrial Biotechnology, Faculty of Biotechnology and Food Sciences, Lodz University of Technology, Stefanowskiego 2/22, 90-537 Łódź, Poland

* Correspondence: anna.podsedek@p.lodz.pl

Table S1. Description of the selected maturity stages for collection of chokeberry fruit

Harvesting date	20th of May	20th of June	20th of July	20th of August
Abbreviation	S1	S2	S3	S4
Maturity stage	unripe	unripe	semi-mature	ripe
Fruit colour	green	green	purple	black
Mass weight (g/100 fruits)	8.81 ± 0.26a	18.57 ± 0.87b	90.88 ± 3.50c	94.43 ± 4.78c
Dry matter (g/100 g of fresh weight)	24.83 ± 0.25b	34.42 ± 0.34c	23.23 ± 0.45a	22.65 ± 0.21a
Fruits at various stages of maturity	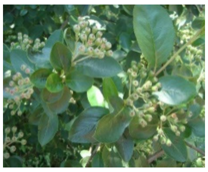	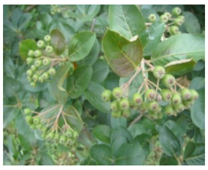	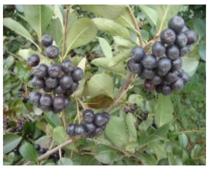	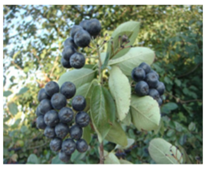
Ground dried fruits	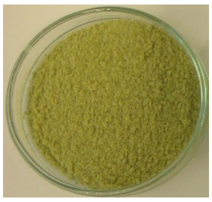	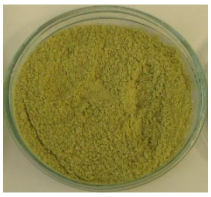	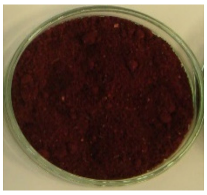	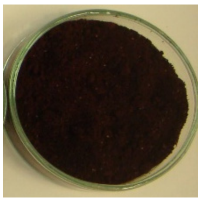

Mean values within a row with different letters are significantly different at $p < 0.05$

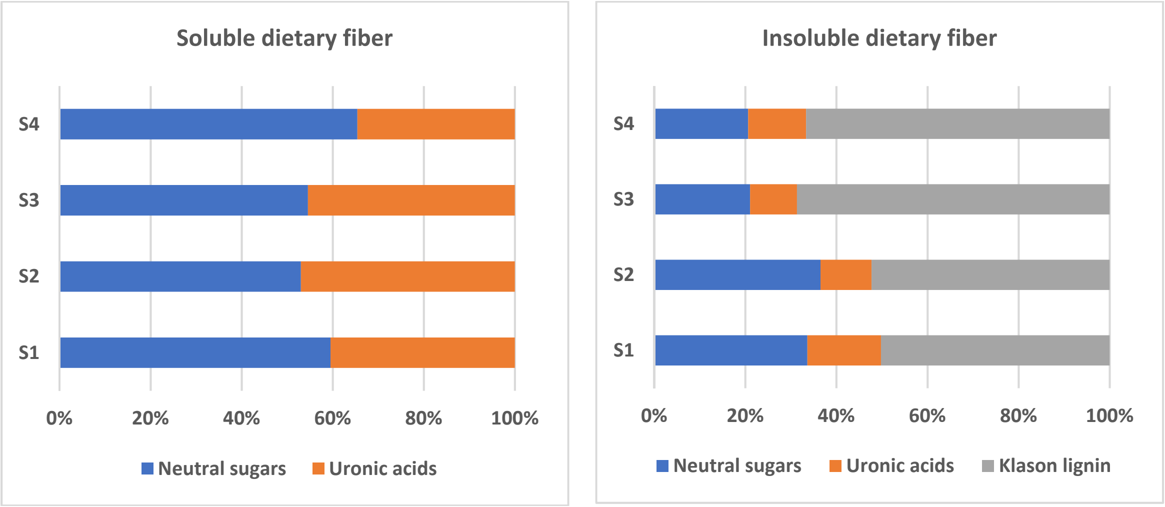

Figure S1. The percentage of soluble and insoluble fiber fraction in chokeberry fruit at various stages of development. S1 – unripe green fruits, harvest May 20; S2 – unripe green fruits, harvest June 20; S3 – semi- mature purple fruits, harvest July 20; S4 – ripe black fruits, harvest August 20.

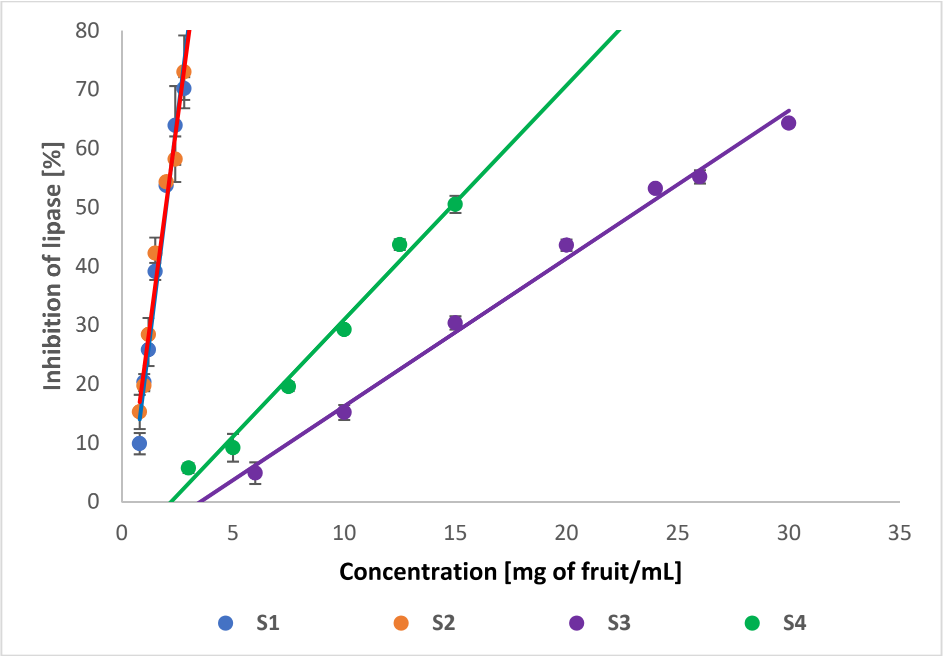

Figure S2. Effect of concentration of chokeberry fruits at various stages of development (S1 – S4) on the inhibition of pancreatic lipase. S1 – unripe green fruits, harvest May 20; S2 – unripe green fruits, harvest June 20; S3 – semi- mature purple fruits, harvest July 20; S4 – ripe black fruits, harvest August 20.

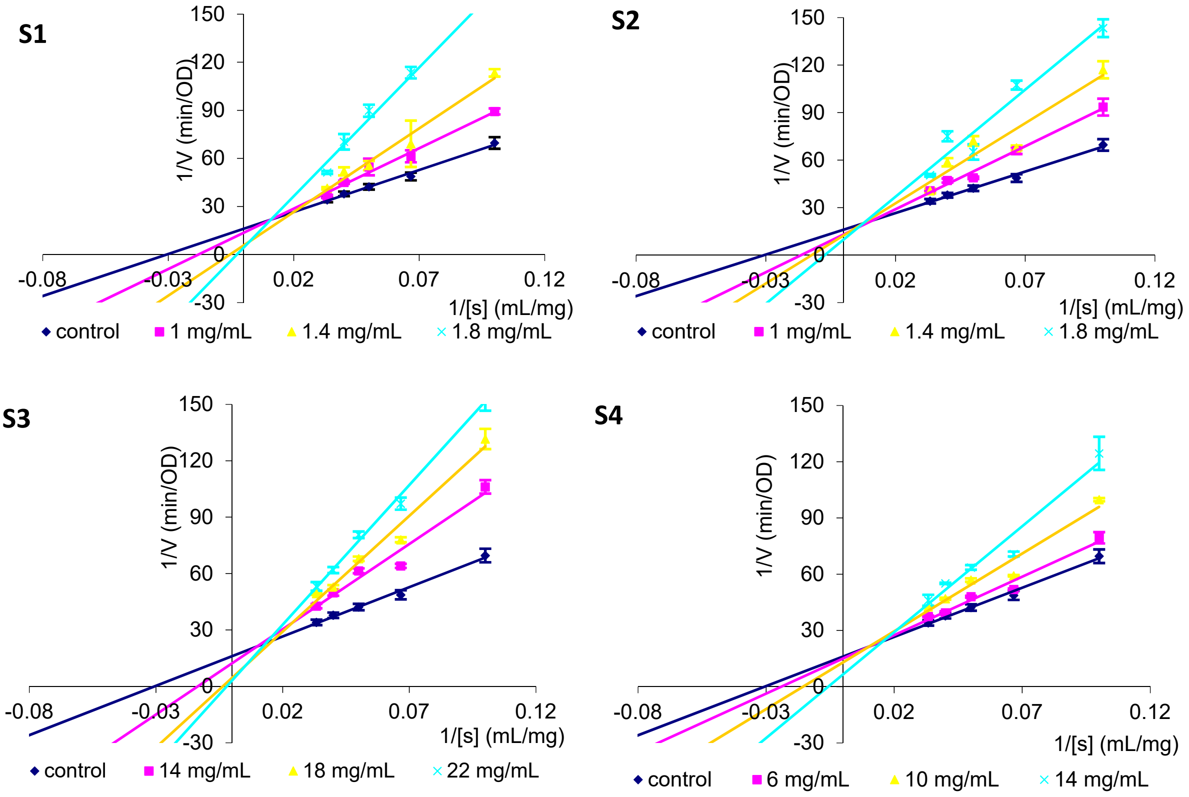

Figure S3. Lineweaver-Burk plots showing the impact of the control (without inhibitor) and chokeberry fruit at various stages of development (S1-S4) on the pancreatic lipase activity; S1 – unripe green fruits, harvest May 20; S2 – unripe green fruits, harvest June 20; S3 – semi- mature purple fruits, harvest July 20; S4 – ripe black fruits, harvest August 20.
